# A Noncanonical DNA Damage Checkpoint Response in a Major Fungal Pathogen

**DOI:** 10.1128/mBio.03044-20

**Published:** 2020-12-15

**Authors:** Erika Shor, Rocio Garcia-Rubio, Lucius DeGregorio, David S. Perlin

**Affiliations:** a Center for Discovery and Innovation, Hackensack Meridian Health, Nutley, New Jersey, USA; b Department of Medical Sciences, Hackensack Meridian School of Medicine, Nutley, New Jersey, USA; c Lombardi Comprehensive Cancer Center, Georgetown University, Washington, DC, USA; Duke University

**Keywords:** *Candida glabrata*, DNA damage checkpoints, DNA damage response, Rad53, cell division

## Abstract

DNA damage checkpoints are key guardians of genome integrity. Eukaryotic cells respond to DNA damage by triggering extensive phosphorylation of Rad53/CHK2 effector kinase, whereupon activated Rad53/CHK2 mediates further aspects of checkpoint activation, including cell cycle arrest and transcriptional changes. Budding yeast Candida glabrata, closely related to model eukaryote Saccharomyces cerevisiae, is an opportunistic pathogen characterized by high genetic diversity and rapid emergence of drug-resistant mutants. However, the mechanisms underlying this genetic variability are unclear. We used Western blotting and mass spectrometry to show that, unlike S. cerevisiae, C. glabrata cells exposed to DNA damage did not induce C. glabrata Rad53 (CgRad53) phosphorylation. Furthermore, flow cytometry analysis showed that, unlike S. cerevisiae, C. glabrata cells did not accumulate in S phase upon DNA damage. Consistent with these observations, time-lapse microscopy showed C. glabrata cells continuing to divide in the presence of DNA damage, resulting in mitotic errors and cell death. Finally, transcriptome sequencing (RNAseq) analysis revealed transcriptional rewiring of the DNA damage response in C. glabrata and identified several key protectors of genome stability upregulated by DNA damage in S. cerevisiae but downregulated in C. glabrata, including proliferating cell nuclear antigen (PCNA). Together, our results reveal a noncanonical fungal DNA damage response in C. glabrata, which may contribute to rapidly generating genetic change and drug resistance.

## Abstract (precis)

In order to preserve genome integrity, all cells must mount appropriate responses to DNA damage, including slowing down or arresting the cell cycle to give the cells time to repair the damage and changing gene expression, for example to induce genes involved in DNA repair. The Rad53 protein kinase is a conserved central mediator of these responses in eukaryotic cells, and its extensive phosphorylation upon DNA damage is necessary for its activation and subsequent activity.

## INTRODUCTION

DNA damage poses an ever-present threat to living cells. Failure to mount an appropriate response to DNA damage can lead to genetic instability, which has a number of biological and pathological consequences, e.g., contributing to the development of cancer and, in microbial pathogens, affecting the evolution of host-pathogen interplay and the emergence of drug-resistant mutants. Fungal DNA damage responses are highly diverse, which has been highlighted by several recent studies showing that genes involved in the maintenance of genome integrity are less conserved than other functional categories of genes in fungal species (1, 2). Indeed, several lineages of yeast genus *Hanseniaspora* lack homologs of dozens of genes involved in chromosome segregation, cell cycle progression, and DNA repair and are characterized by higher evolution rates, at least in the beginning of their lineage’s evolution (1). The genetic instability of fungal pathogens, which is of particular interest due to its relevance to evolution of drug resistance, has been extensively reported, particularly during host colonization or under stressful environmental conditions (3–8). The types of genetic alterations most commonly described in fungi are aneuploidies and loss of heterozygosity, which occur in diploid or polyploid fungi, such as Candida albicans or Cryptococcus neoformans (9). Although haploid fungi cannot avail themselves of these mechanisms, they can also exhibit extensive genetic variation and rapid emergence of drug-resistant mutants (10–14), suggesting the existence of other, as yet unknown, mechanisms that enable high genetic “flexibility” in fungal pathogens.

Candida glabrata is a haploid budding yeast more closely related to baker’s yeast Saccharomyces cerevisiae than to C. albicans (15). Unlike S. cerevisiae, however, C. glabrata is an obligate human commensal microbe that can become pathogenic and is a leading cause of life-threatening invasive fungal infections in immunocompromised individuals (16–18). C. glabrata rapidly evolves resistance to different antifungal drug classes (14, 19–23) and is characterized by extremely high genome variation among clinical isolates both in terms of single nucleotide polymorphisms and larger structural variants (11–14, 24). The documented extensive chromosomal variation among C. glabrata clinical isolates resembles the unstable karyotypes and increased gross chromosomal rearrangements observed in S. cerevisiae mutants lacking DNA replication checkpoint functions (25, 26). However, checkpoint activity in C. glabrata has not been examined.

Response to DNA damage depends on the cell cycle phase, but damage incurred during the process of DNA replication is considered to be especially detrimental as it can lead to replication fork destabilization, the formation of double-strand breaks at collapsed forks, and inappropriate recombination, resulting in chromosomal rearrangements and cell death (27, 28). DNA replication checkpoint slows down S-phase progression, stabilizes replication forks, inhibits replication origin firing, and upregulates transcription of DNA repair genes (29–32). Cells with unrepaired DNA damage at the end of S phase also activate the G_2_/M checkpoint, which arrests cells in mitosis (33, 34). Although these checkpoints differ with regard to their specific triggers, protein players, and downstream effects, several proteins play key roles in both checkpoints, most notably the Rad53 serine/threonine kinase (CHK2 in higher eukaryotes). Rad53 is a checkpoint effector kinase—upon DNA damage or DNA replication arrest, it is extensively phosphorylated by upstream sensor kinases and by itself (35, 36), thereupon amplifying the DNA damage signal by phosphorylating dozens of downstream targets (37–39). Rad53 phosphorylation is instrumental for virtually all aspects of the DNA damage response (38, 40–44). Rad53 orthologs are also extensively phosphorylated upon DNA damage in several non-*Saccharomyces* fungal species examined, including C. albicans, C. neoformans, and Schizosaccharomyces pombe (45–47). However, the phosphorylation of Rad53 in C. glabrata has not been studied.

In this study, we examined the DNA damage response of C. glabrata, focusing on Rad53 phosphorylation, cell cycle alterations, and the global transcriptomic response. Interestingly, we did not detect a DNA damage-induced increase in Rad53 phosphorylation in C. glabrata. Consistent with this finding, in the presence of DNA damage, C. glabrata cells did not accumulate in S phase and proceeded to divide, giving rise to mitotic errors and significant cell death. Finally, using transcriptome sequencing (RNAseq), we obtained evidence of transcriptional rewiring of the DNA damage response in C. glabrata, as well as differential regulation of several key protectors of genome integrity, including proliferating cell nuclear antigen (PCNA). Together, these results reveal previously unappreciated variation in fungal DNA damage responses and have important implications for fungal genome stability, evolution, and emergence of antifungal drug resistance.

## RESULTS

### C. glabrata does not induce CgRad53 phosphorylation upon DNA damage.

**FIG 1:**
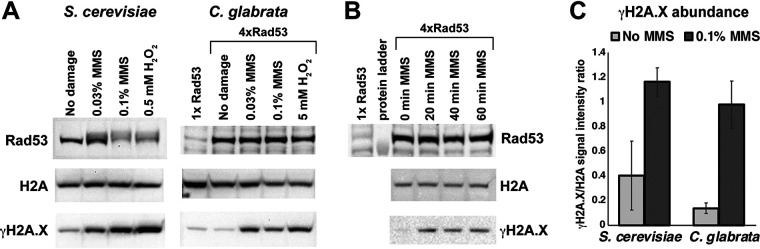
DNA damage induced a change in Rad53 mobility in S. cerevisiae but not C. glabrata. (A) Alkylating damage (MMS) and oxidative damage (H_2_O_2_) induced a shift in the mobility of ScRad53 but not CgRad53. Both conditions induced DNA damage in both species, as evidenced by increased abundance of γH2A.X. (B) MMS induced an increase in γH2A.X abundance by 20 min postexposure but did not induce even a transient shift in CgRad53 mobility. (C) MMS treatment induced DNA damage, as reflected by γH2A.X levels, to similar extents in S. cerevisiae and C. glabrata. Results were calculated from at least three independent biological replicates for every condition. In panels A and C, the cells were exposed to the indicated DNA damaging agent for 1 h.

To begin to elucidate the role of the DNA damage checkpoint in C. glabrata, we examined the phosphorylation of C. glabrata Rad53 (CgRad53; encoded by *CAGL0M02233g*). Rabbit polyclonal antibodies raised against short peptides in the CgRad53 N and C termini did not efficiently detect endogenous CgRad53, but adding a plasmid-borne copy of the gene driven by a weak promoter (48) resulted in a fourfold overexpression of *CgRAD53* (see Fig. S1 in the supplemental material) and robust detection of the protein, allowing us to examine its mobility on SDS-PAGE in the absence and presence of DNA damage. As a control, S. cerevisiae Rad53 (ScRad53) was examined as well. Consistent with existing literature, we detected a shift in ScRad53 mobility upon exposure to the DNA alkylating agent methyl methanesulfonate (MMS) and oxidative damage by H_2_O_2_ (Fig. 1A), reflecting extensive phosphorylation. In contrast, we did not detect a mobility shift for CgRad53, either in MMS or in H_2_O_2_ (Fig. 1A). We considered the possibility that CgRad53 phosphorylation occurred rapidly and transiently, so we examined CgRad53 mobility starting 20 min after the addition of MMS; however, no mobility shift was detected (Fig. 1B). To confirm that C. glabrata was experiencing DNA damage in the presence of MMS or H_2_O_2_, we measured the abundance of histone H2A phosphorylated at serine 129 (also known as γH2A.X), a universal marker of DNA damage, particularly double-strand breaks (49). We found that γH2A.X was strongly induced both by MMS and by H_2_O_2_ in C. glabrata (Fig. 1A and B). Consistent with reports that C. glabrata is highly resistant to oxidative damage (50), it required a much higher concentration of H_2_O_2_ than S. cerevisiae to cause significant DNA damage (Fig. 1A). However, the effects of MMS on γH2A.X levels in S. cerevisiae and in C. glabrata were similar (Fig. 1C). Together, these data indicated that despite efficient induction of DNA damage in C. glabrata, CgRad53 mobility did not change, indicating that extensive phosphorylation was not occurring.

10.1128/mBio.03044-20.1 FIG S1 *CgRAD53* was mildly overexpressed by placing it downstream of the p*EGD2* promoter. *CgRAD53* was inserted downstream of p*EGD2* (a weak promoter) on a plasmid (48) and transformed into wild-type C. glabrata (strain ATCC 2001). Quantitative PCR (qPCR) (primers GAGGATTTACAGGTGCTACAGG and CGACCACATATCAACTAGGGAAG) was used to measure the level of *CgRAD53* expression in cells carrying p*EGD2-CgRAD53* relative to cells carrying the empty vector. *CgPGK1* gene was used as a control locus. *N* = 3 biological replicates. Download FIG S1, TIF file, 0.8 MB. Copyright © 2020 Shor et al. 2020 Shor et al. This content is distributed under the terms of the Creative Commons Attribution 4.0 International license.

**FIG 2:**
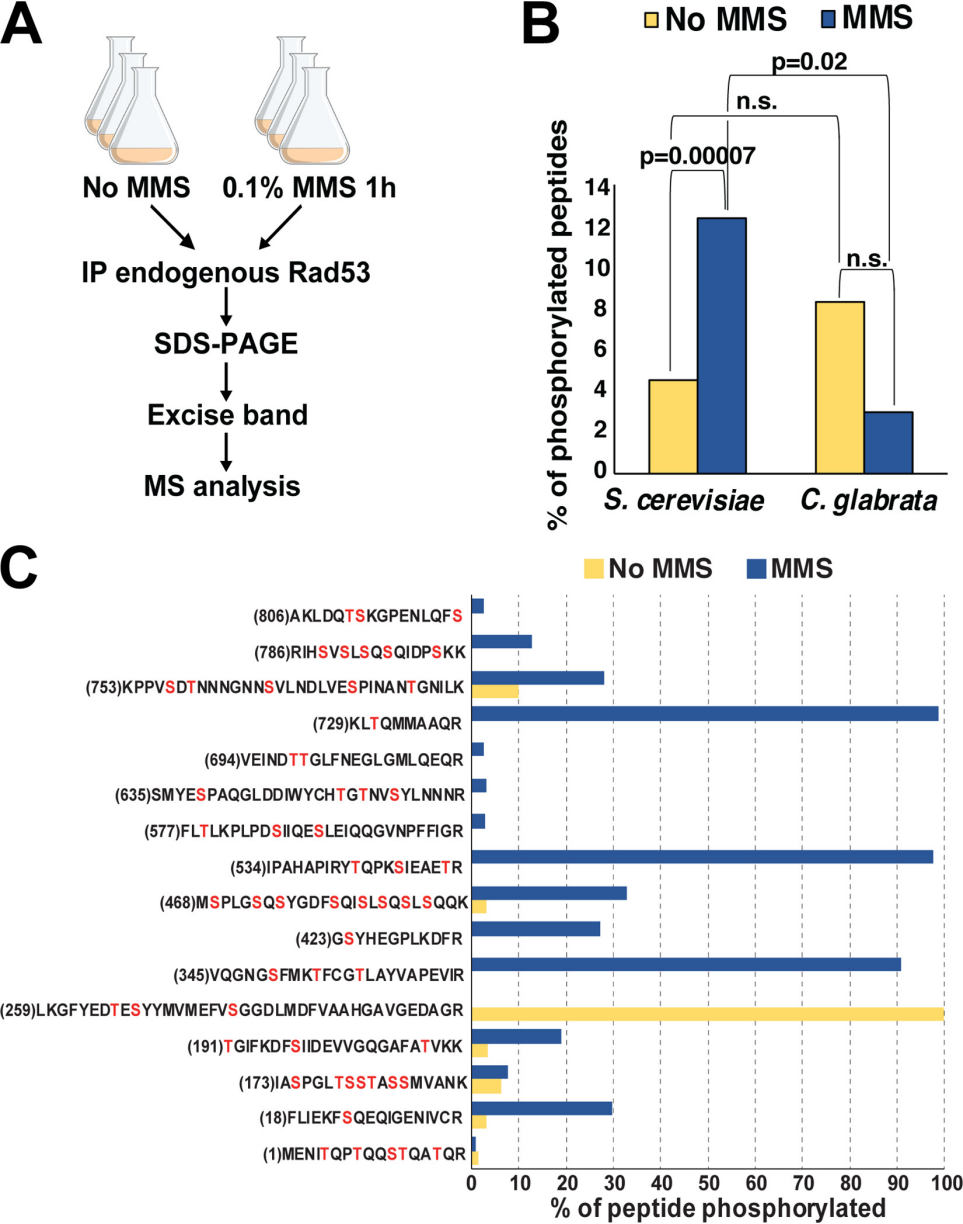
Mass spectrometry (MS) analysis detected extensive DNA damage-induced Rad53 phosphorylation in S. cerevisiae but not C. glabrata. (A) Outline of the experiment. IP, immunoprecipitate. (B) The fraction of phosphorylated Rad53 peptides was significantly increased in S. cerevisiae samples, but not C. glabrata samples, derived from MMS-treated cells. The *P* value was calculated using the χ^2^ test. n.s., not significant. (C) Consistent with previous studies, our MS analysis identified extensive DNA damage-induced phosphorylation throughout ScRad53. For each peptide, the total intensity of the phosphorylated forms of that peptide was divided by the total intensity of all forms of that peptide, converted to percentages, and plotted on the *y* axis. The number in parentheses indicates the position of the first residue in the peptide. Serines and threonines are shown in red.

To further investigate the phosphorylation status of CgRad53 in the absence and presence of DNA damage, we immunoprecipitated endogenous CgRad53 and ScRad53 from untreated and MMS-treated C. glabrata and S. cerevisiae, respectively, and subjected them to mass spectrometry (MS) analysis (Fig. 2A). We identified 346 and 451 unique ScRad53 peptides isolated from untreated and MMS-treated cells, respectively, corresponding to 75% and 82% protein coverage (see Data Set S1 and Fig. S2 in the supplemental material). For CgRad53, we identified 45 and 63 unique peptides obtained from untreated and MMS-treated cells, respectively, corresponding to 44% and 49% protein coverage (Data Set S1 and Fig. S2). Consistent with existing literature, we detected a strong increase in the fraction of ScRad53 phosphopeptides in the MMS-treated sample (Fig. 2B and C and Data Set S1). In contrast, and consistent with the Western blot data (Fig. 1A and B), the fraction of CgRad53 phosphopeptides did not increase after MMS treatment (Fig. 2B and Data Set S1), supporting the conclusion that C. glabrata Rad53 was not significantly phosphorylated upon DNA damage.

10.1128/mBio.03044-20.2 FIG S2 Peptides identified by MS in ScRad53 and CgRad53 (shown in green). Download FIG S2, TIF file, 2.8 MB. Copyright © 2020 Shor et al. 2020 Shor et al. This content is distributed under the terms of the Creative Commons Attribution 4.0 International license.

10.1128/mBio.03044-20.7 DATA SET S1 This data set contains processed data obtained by MS (the first sheet of the Excel file) and by RNAseq (the other sheets). Download Data Set S1, XLSX file, 1.5 MB. Copyright © 2020 Shor et al. 2020 Shor et al. This content is distributed under the terms of the Creative Commons Attribution 4.0 International license.

To identify the protein features that may contribute to this lack of phosphorylation, we scrutinized the CgRad53 amino acid sequence. CgRad53 is slightly shorter than ScRad53 (767 versus 821 amino acids), but its overall domain organization is similar to that of ScRad53, containing a kinase domain flanked by two FHA domains (Fig. S3A). Likewise, the two proteins contain a similar percentage of serines and threonines (Fig. S3B). However, an examination of the ScRad53-CgRad53 protein alignment revealed that a number of serines and threonines phosphorylated in ScRad53 were not conserved in CgRad53 (Fig. S3C). Interestingly, most ScRad53 S/TQ motifs, which are canonical phosphorylation sites for phosphatidylinositol 3-kinase (PI3-K)-related kinases, such as Mec1 and Tel1, are conserved in CgRad53, with the exception of Ser53, Thr731, and Ser795. In contrast, all three ScRad53 proline-directed phosphorylation sites (Ser175, Ser375, and Ser774), which are phosphorylated by cyclin-dependent kinases (51, 52), are not conserved in CgRad53 (Fig. S3C). Likewise, a number of noncanonical (non-S/TQ) Mec1 sites and ScRad53 autophosphorylation sites are not conserved in CgRad53 (Fig. S3C). Importantly, the majority of serines and threonines phosphorylated in ScRad53 but lacking conservation in CgRad53 have been shown to be targets of MMS-induced phosphorylation (Fig. S3C). This lack of conservation, together with the results shown above, supports the conclusion that in C. glabrata, Rad53 is not targeted for extensive DNA damage-induced phosphorylation.

10.1128/mBio.03044-20.3 FIG S3 **(**A) Overall Rad53 domain organization is similar in S. cerevisiae and C. glabrata. (B) The overall percentages of serines and threonines are similar in ScRad53 and CgRad53. (C) The Rad53 alignment between S. cerevisiae and C. glabrata orthologs was performed using MUSCLE in MegAlign Pro software (Lasergene). ScRad53 phosphosite data were obtained from references 35, 51, 52, 62, and 90. Download FIG S3, TIF file, 2.8 MB. Copyright © 2020 Shor et al. 2020 Shor et al. This content is distributed under the terms of the Creative Commons Attribution 4.0 International license.

### C. glabrata cells do not accumulate in S phase upon DNA damage.

**FIG 3:**
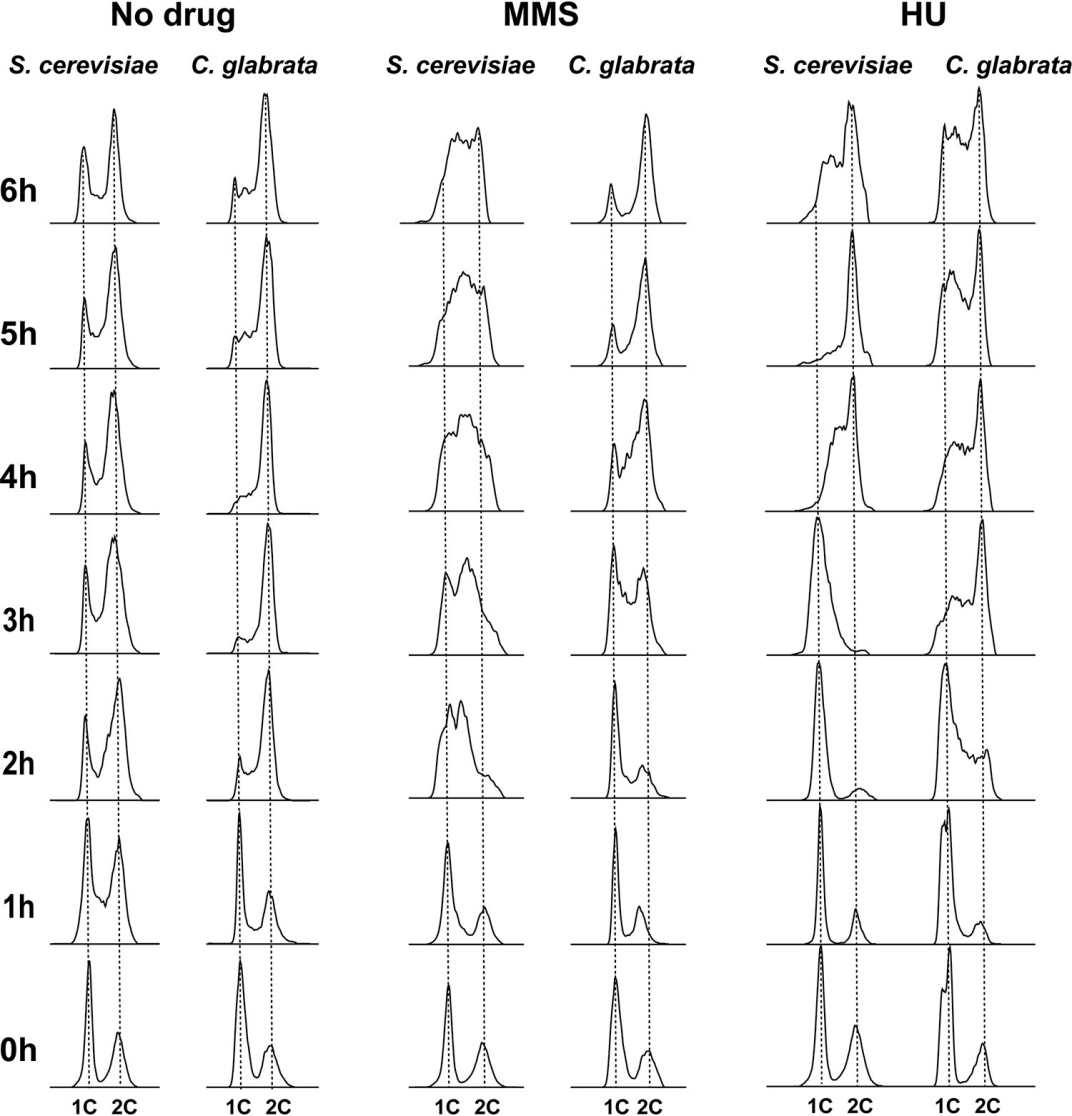
DNA damage induced significant S-phase accumulation in S. cerevisiae but not in C. glabrata. S. cerevisiae and C. glabrata cells were synchronized in G_1_ phase by carbon starvation and released into glucose-containing medium either in the absence or presence of MMS (0.03%) or HU (100 mM). In the presence of MMS, C. glabrata completed DNA replication much faster than S. cerevisiae cells, which remained accumulated in S phase by the end of the 6-h time course. SYTOX green staining and flow cytometry were used to measure DNA content.

A key consequence of DNA damage signaling replication checkpoint activation via Rad53 phosphorylation is the slowing of DNA replication, which allows cells time to repair the damage prior to cell division (53). A typical method of detecting this in S. cerevisiae involves synchronizing cells in G_1_ with α-factor and releasing them into DNA damaging conditions. Because C. glabrata cells do not arrest in response to mating pheromones (54), we used carbon starvation to synchronize C. glabrata and S. cerevisiae cells in G_1_ (Fig. 3). The synchronized cells were then released into glucose-containing medium in the absence or presence of 0.03% MMS, and cell cycle distribution was analyzed for 6 h by flow cytometry. Hydroxyurea (HU) (100 mM), which inhibits DNA replication by depleting deoxynucleoside triphosphate (dNTP) pools but without inducing DNA damage, was used as a comparator. Consistent with previous reports (53, 55, 56), S. cerevisiae cells released into MMS-containing medium significantly slowed down DNA replication, remaining largely accumulated in S phase by the end of the 6 h (Fig. 3). In contrast, while C. glabrata cells were slowed down by the presence of MMS in terms of their entry into S phase (compare 2-h time points for “No drug” and “MMS” in Fig. 3), they did not accumulate in S phase and largely completed DNA replication between 4 and 5 h after MMS exposure (Fig. 3). We did this experiment at both 30°C and 37°C (the optimal C. glabrata growth temperature) and obtained identical results (Fig. S4). Finally, C. glabrata released in the presence of HU also delayed the start of DNA replication, but unlike in the presence of MMS, did not complete it by the end of the 6-h period, at which point a large proportion of the population still remained in S phase (Fig. 3), whereupon their cell cycle profiles looked similar to HU-exposed S. cerevisiae cells (Fig. 3). Together, these data show that activation of the S-phase checkpoint by DNA damage (but not by non-damage-associated inhibition of DNA replication) is significantly attenuated in C. glabrata compared to S. cerevisiae.

**FIG 4:**
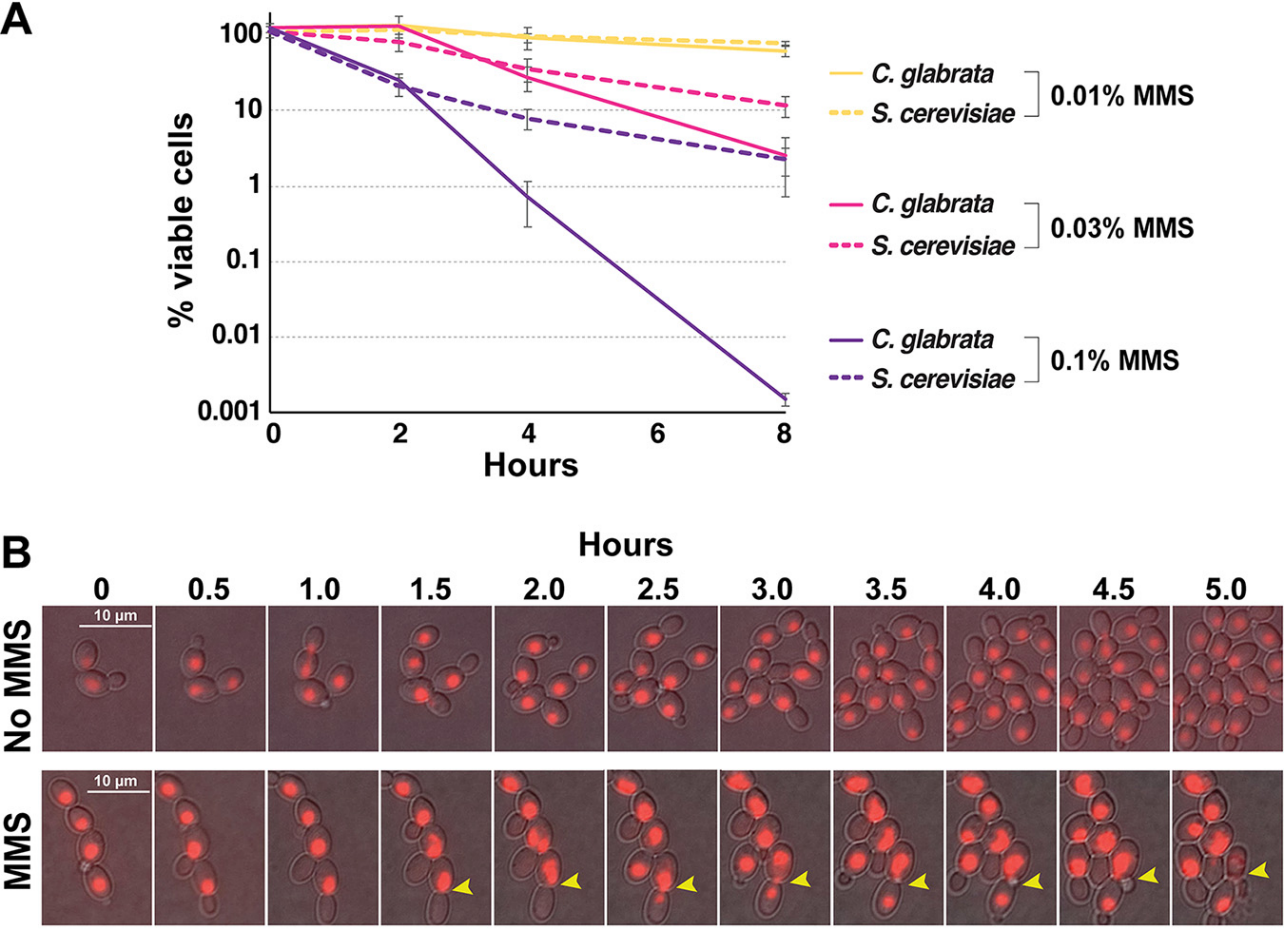
C. glabrata exhibited high lethality and aberrant mitoses in the presence of DNA damage. (A) C. glabrata cells are more sensitive to high levels of DNA damage than S. cerevisiae cells. Cells were cultured in the presence of indicated concentrations of MMS, harvested at the indicated time points, counted, and plated on drug-free YPD plates. Viability counts were obtained by dividing the number of resulting colonies by the number of plated cells. Results were calculated from at least three biological replicates for every time point. (B) Time-lapse microscopy detected C. glabrata cells dividing in the presence of 0.03% MMS, including aberrant nuclear divisions. The cells, carrying an NLS-RFP construct to fluorescently mark nuclei, were pipetted onto YPD-agarose pads, sealed, and imaged for 6 h at 10-min intervals. Thirty-minute intervals are shown. Yellow arrowheads indicate cells where nuclear material was unequally distributed into mother and daughter cells. Both mother and daughter cells subsequently budded, but the mother cell burst. The corresponding time-lapse movies are available as Movies S1 and S2 in the supplemental material.

10.1128/mBio.03044-20.4 FIG S4 C. glabrata cells synchronized in G_1_ by carbon starvation and released into 0.03% MMS or 100 mM HU at 30°C behaved similarly to cells grown and released into these drugs at 37°C (Fig. 4). Download FIG S4, TIF file, 0.9 MB. Copyright © 2020 Shor et al. 2020 Shor et al. This content is distributed under the terms of the Creative Commons Attribution 4.0 International license.

### C. glabrata cells undergo aberrant cell divisions and lose viability in response to DNA damage.

In S. cerevisiae, Rad53-mediated checkpoint signaling is essential for surviving DNA damage, wherein *rad53* mutants (both deletion and point mutants lacking phospho-sites) and other checkpoint mutants proceed with the cell cycle in the presence of DNA damaging agents and exhibit high lethality, presumably due to aberrant replication and division (56). Because C. glabrata exhibited highly attenuated DNA damage-induced Rad53 phosphorylation and checkpoint activation, we measured its ability to survive DNA damage. We found that whereas at a low concentration of MMS (0.01%), viability was moderately and similarly impacted in S. cerevisiae and C. glabrata, at higher MMS concentrations (0.03% and 0.1%), C. glabrata was significantly more sensitive than S. cerevisiae, exhibiting several orders of magnitude higher lethality after 8 h in 0.1% MMS (Fig. 4A).

10.1128/mBio.03044-20.8 MOVIE S1 This video shows a 6-h time-lapse movie (imaged at 10-min intervals) of C. glabrata cells growing in YPD-low-melting-point agarose in the absence of MMS. Nuclei are marked with NLS-RFP. Download Movie S1, MOV file, 0.09 MB. Copyright © 2020 Shor et al. 2020 Shor et al. This content is distributed under the terms of the Creative Commons Attribution 4.0 International license.

10.1128/mBio.03044-20.9 MOVIE S2 This video shows a 6-h time-lapse movie (imaged at 10-min intervals) of C. glabrata cells growing in YPD-low-melting-point agarose in the presence of 0.03% MMS. Nuclei are marked with NLS-RFP. Download Movie S2, MOV file, 0.2 MB. Copyright © 2020 Shor et al. 2020 Shor et al. This content is distributed under the terms of the Creative Commons Attribution 4.0 International license.

To gain insight into the causes of lethality in MMS-treated C. glabrata cells, we used time-lapse microscopy to track cell division of C. glabrata cells with fluorescently marked nuclei (NLS-RFP; see Movies S1 and S2 in the supplemental material). We observed that whereas the presence of 0.03% MMS significantly slowed down the rate at which new buds emerged, nevertheless, 2 to 3 h after the addition of MMS, a number of cells proceeded with nuclear division and mitosis (Fig. 4B and Movies S1 and S2). Furthermore, we were able to observe aberrant mitoses wherein nuclear content was distributed unequally between mother and daughter cells prior to cytokinesis (Fig. 4B, yellow arrowheads). Despite this unequal distribution of nuclear content, both mother and daughter cells proceeded to bud; however, the mother cell subsequently “exploded” (Fig. 4B, yellow arrowheads). To track how often such catastrophic cell divisions occur, we counted cell deaths (determined by visible loss of cellular or nuclear integrity) upon cell division either in the presence or absence of MMS. In the absence of MMS, we tracked 464 divisions and observed only two deaths, whereas in the presence of MMS, we tracked 60 division events and observed five deaths (0.43% versus 8.3%, respectively; χ^2^ *P* value < 0.00001). These observations, together with the cell cycle distribution analysis (Fig. 3) and cell viability measurements (Fig. 4A), show that C. glabrata cells do not significantly activate the DNA damage checkpoint, that many of them proceed with S phase and cell division even in the presence of DNA damage, and as a result lose viability due to aberrant mitoses.

### A rewiring of the transcriptional response to DNA damage in C. glabrata.

**FIG 5:**
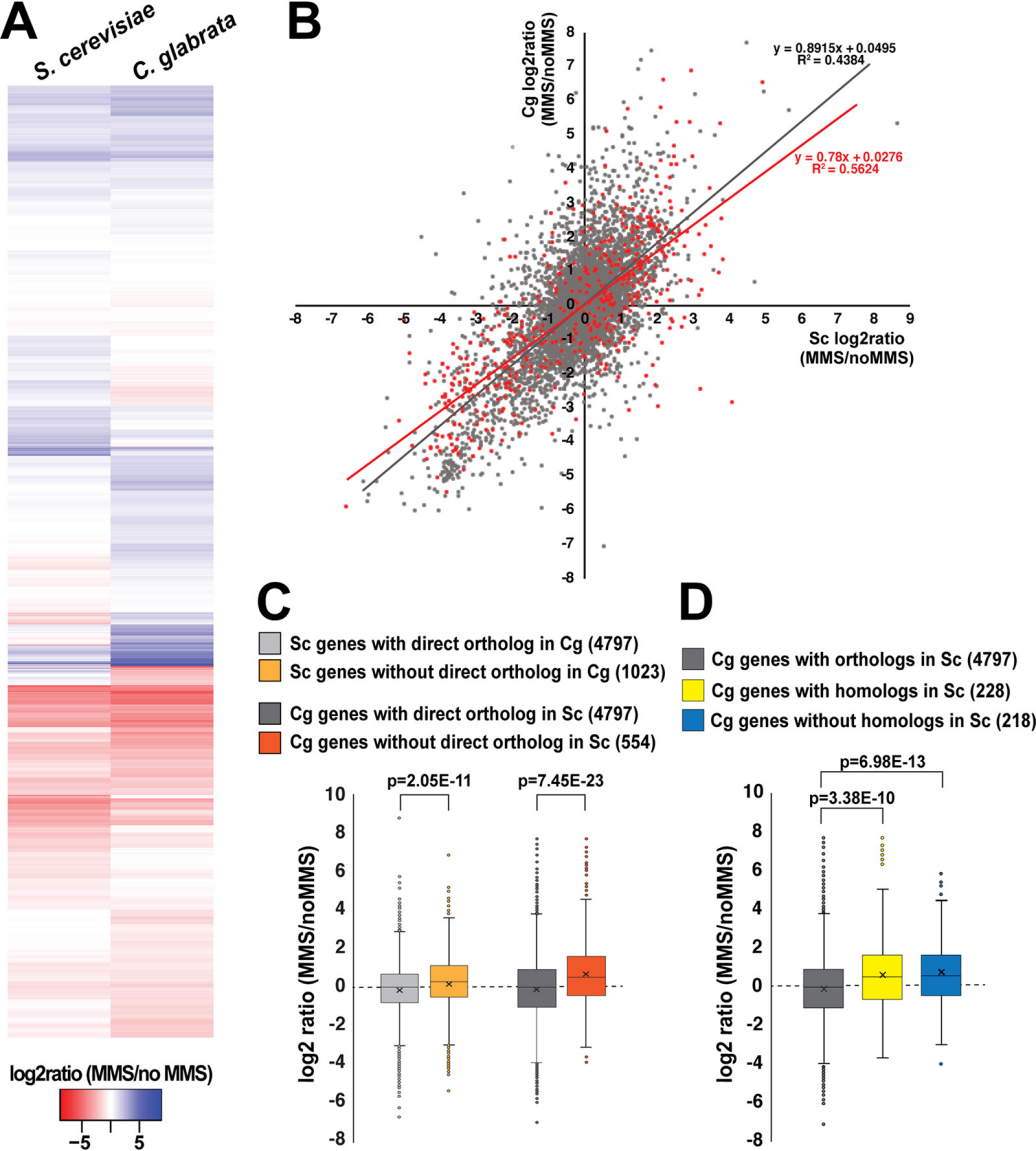
RNAseq revealed evidence of transcriptional rewiring of the DNA damage response in C. glabrata relative to S. cerevisiae. C. glabrata and S. cerevisiae cells were treated with 0.1% MMS for 1 h, and then total RNA was isolated from both untreated and treated cells and subjected to RNAseq analysis. Three biological replicates of every condition were analyzed, with the exception of “S. cerevisiae no MMS” for which one of the samples had poor RNA quality and was not processed further. (A) Heatmap of the “MMS/no MMS” log_2_ ratios for S. cerevisiae genes and their C. glabrata orthologs. (B) Scatterplot where each gene is represented by a dot and its “MMS/no MMS” log_2_ ratio for S. cerevisiae (Sc) is plotted on the *x* axis and the ratio for C. glabrata (Cg) is plotted on the *y* axis. Genes whose expression is regulated by Rad53 in S. cerevisiae are indicated in red. (C) In both C. glabrata and S. cerevisiae, genes that lack a direct ortholog in the other species are induced more strongly by DNA damage. (D) Both C. glabrata genes that have homologs but not direct orthologs in S. cerevisiae and C. glabrata genes that have no homologs in S. cerevisiae tend to be upregulated by MMS. The *P* values were calculated by an unpaired two-tailed *t* test. The S. cerevisiae-C. glabrata ortholog list was downloaded from http://www.candidagenome.org/download/homology/orthologs. In panels C and D, the number in parentheses indicates the number of genes in the corresponding category.

A key part of the cellular response to DNA damage is activated Rad53 phosphorylating multiple transcription factors, which in turn alter the expression of hundreds of genes, e.g., downregulating genes involved in growth and cell cycle progression and upregulating genes involved in stress responses and DNA repair (38, 43). To ask whether a similar transcriptional response exists in C. glabrata, we cultured both C. glabrata and S. cerevisiae in the absence or presence of 0.1% MMS for 1 h, isolated total RNA, and analyzed it by transcriptome sequencing (RNAseq). As reported previously, over 2,000 genes were up- or downregulated by DNA damage in S. cerevisiae (Fig. 5A and Data Set S1), and these transcriptional changes were consistent with those published previously (Fig. S5A) (38, 43). Over 2000 genes were also up- and downregulated by DNA damage in C. glabrata (Fig. 5A), and interestingly, there was a high degree of concordance between the expression changes of orthologous genes present in both species (4,797 genes; Fig. 5A and B). This concordance was especially strong for genes downregulated by MMS: in both S. cerevisiae and C. glabrata, these genes were strongly enriched for those involved in protein synthesis, e.g., translation, ribosome biogenesis, and rRNA processing (Fig. S6A). This downregulation of progrowth genes was consistent with previous reports (38, 43) and with our conclusion that in the presence of MMS C. glabrata was experiencing DNA damage-induced stress. Interestingly, gene categories induced by MMS were more diverse in C. glabrata than in S. cerevisiae. Both species induced genes involved in protein degradation and stress responses; however, C. glabrata also induced orthologs of genes, which in S. cerevisiae are involved in sporulation and meiosis (Fig. S6A). This observation was intriguing and unexpected because mating and meiosis have not been detected in C. glabrata to date.

10.1128/mBio.03044-20.5 FIG S5 Comparing our RNAseq data to other data sets. (A) The plots show correlations between our S. cerevisiae RNAseq data (log_2_ ratio of MMS/noMMS for every gene) with those obtained in references 38 and 43. The differences with our data are likely due to the fact that those studies used lower concentrations of MMS (0.02% or 0.03% versus the 0.1% that we used) and because they were performed using microarray technology. (B) This panel shows a comparison of transcriptional responses to MMS of genes lost from *Hanseniaspora* yeast lineages. Out of all genes lost from *Hanseniaspora*, genes involved in chromosomal maintenance (cell cycle regulation, chromosome segregation, and DNA repair) were more likely to show divergent responses to MMS in S. cerevisiae and C. glabrata than genes involved in other processes. Download FIG S5, TIF file, 0.6 MB. Copyright © 2020 Shor et al. 2020 Shor et al. This content is distributed under the terms of the Creative Commons Attribution 4.0 International license.

10.1128/mBio.03044-20.6 FIG S6 Gene Ontology (GO) analysis of genes upregulated or downregulated by MMS in S. cerevisiae and C. glabrata. (A) Genes upregulated or downregulated at least twofold in the presence of 0.1% MMS were subjected to GO analysis using the FungiFun tool (https://elbe.hki-jena.de/fungifun/) (89). (B) Genes that were differentially regulated by MMS in S. cerevisiae and C. glabrata (i.e., genes whose log_2_ MMS/noMMS ratio differed by at least 2, reflecting at least a fourfold difference in the increase or decrease of transcript abundance in MMS between S. cerevisiae and C. glabrata) were subjected to GO analysis using the FungiFun tool (https://elbe.hki-jena.de/fungifun/) (89). Download FIG S6, TIF file, 1.5 MB. Copyright © 2020 Shor et al. 2020 Shor et al. This content is distributed under the terms of the Creative Commons Attribution 4.0 International license.

We also specifically examined genes whose DNA damage-induced expression changes (either up- or downregulation) in S. cerevisiae are known to be Rad53 dependent (38). Interestingly, the majority of these genes’ orthologs were also responsive to DNA damage in C. glabrata, and their overall response to MMS was similar in C. glabrata to that in S. cerevisiae (Fig. 5B, red dots). This result suggested that this set of genes had undergone transcriptional “rewiring” in C. glabrata, whereby their transcription was robustly induced or repressed by DNA damage within the time frame (1 h) where CgRad53 phosphorylation was not induced and that therefore these changes may have been mediated by factors other than Rad53.

We also individually examined several canonical Rad53 target genes, i.e., those whose transcription has long been known to be strongly induced by DNA damage in a Rad53-dependent manner, specifically ribonucleotide reductase subunit *RNR3* and ribonucleotide reductase inhibitor *HUG1* (57, 58). However, we found that the C. glabrata genome did not contain direct orthologs of either S. cerevisiae *RNR3* (*ScRNR3*) or *ScHUG1*. This prompted us to compare DNA damage-induced transcriptional changes of genes that had a direct ortholog in the other species (∼73% of all S. cerevisiae genes) to those that lacked such orthologs. The S. cerevisiae-C. glabrata ortholog information was obtained from the *Saccharomyces* Genome Database (www.yeastgenome.org) and the *Candida* Genome Database (www.candidagenome.org). Interestingly, we found that in both S. cerevisiae and C. glabrata, genes that lacked a direct ortholog in the other species were significantly more likely to be induced by DNA damage than genes that did have an ortholog (Fig. 5C). To probe this phenomenon further, we focused on the genes in C. glabrata that according to www.candidagenome.org did not have a direct ortholog in S. cerevisiae. These genes generally could be subdivided into two categories: those that had homologs in S. cerevisiae and those for which BLAST searches had revealed no homologs in S. cerevisiae (Data Set S1). Interestingly, we found that both groups tended to be significantly more induced by MMS than genes with direct orthologs in S. cerevisiae (Fig. 5D). In particular, we identified 28 C. glabrata genes lacking identifiable S. cerevisiae homologs that were at least twofold downregulated by MMS and 77 such genes that were at least twofold upregulated by MMS (Data Set S1). These results suggested that the transcriptional response to DNA damage has been diverging in S. cerevisiae and C. glabrata during evolution, consistent with other evidence of transcriptional rewiring.

Finally, we asked whether genes lost from several *Hanseniaspora* yeast lineages, which are enriched for those involved in various genome integrity maintenance functions (1), tend to show divergent transcriptional responses to MMS in S. cerevisiae and C. glabrata. We sourced the list of 950 genes lost from at least one *Hanseniaspora* lineage from reference 1 and examined their responses to MMS in S. cerevisiae and C. glabrata (Data Set S1) . Taken as a whole, these genes showed a robust correlation between the two yeasts (Fig. S5B), although not as strong as the one between the entire sets of orthologous genes (Fig. 5B). We also identified 131 genes lost from *Hanseniaspora* that are known to be involved in various aspects of chromosome maintenance (predominantly chromosome segregation, cell cycle regulation, and DNA repair). Interestingly, the correlation between their transcriptional responses to MMS in S. cerevisiae and C. glabrata was virtually abolished (Fig. S5B), showing that these genes, which are under relaxed selection in yeast (1), also may not show conserved transcriptional regulation by DNA damage.

### DNA damage differentially regulates the expression of proliferating cell nuclear antigen (PCNA) in C. glabrata and S. cerevisiae.

As is evident from Fig. 5A, a number of genes were differentially regulated in S. cerevisiae and C. glabrata. We defined “differential regulation” as a difference of at least 2 log units, or fourfold, in expression change. For instance, by this criterion, a gene whose expression was unchanged by MMS in S. cerevisiae would be considered differentially regulated in C. glabrata if its expression was induced or repressed at least fourfold in that organism. Genes that were upregulated by MMS in C. glabrata relative to S. cerevisiae were enriched for sulfate assimilation (likely in response to MMS), certain types of amino acid metabolism, and meiosis, whereas genes that were downregulated by MMS in C. glabrata relative to S. cerevisiae were enriched for nucleotide/nucleoside metabolism (Fig. S6B).

**FIG 6:**
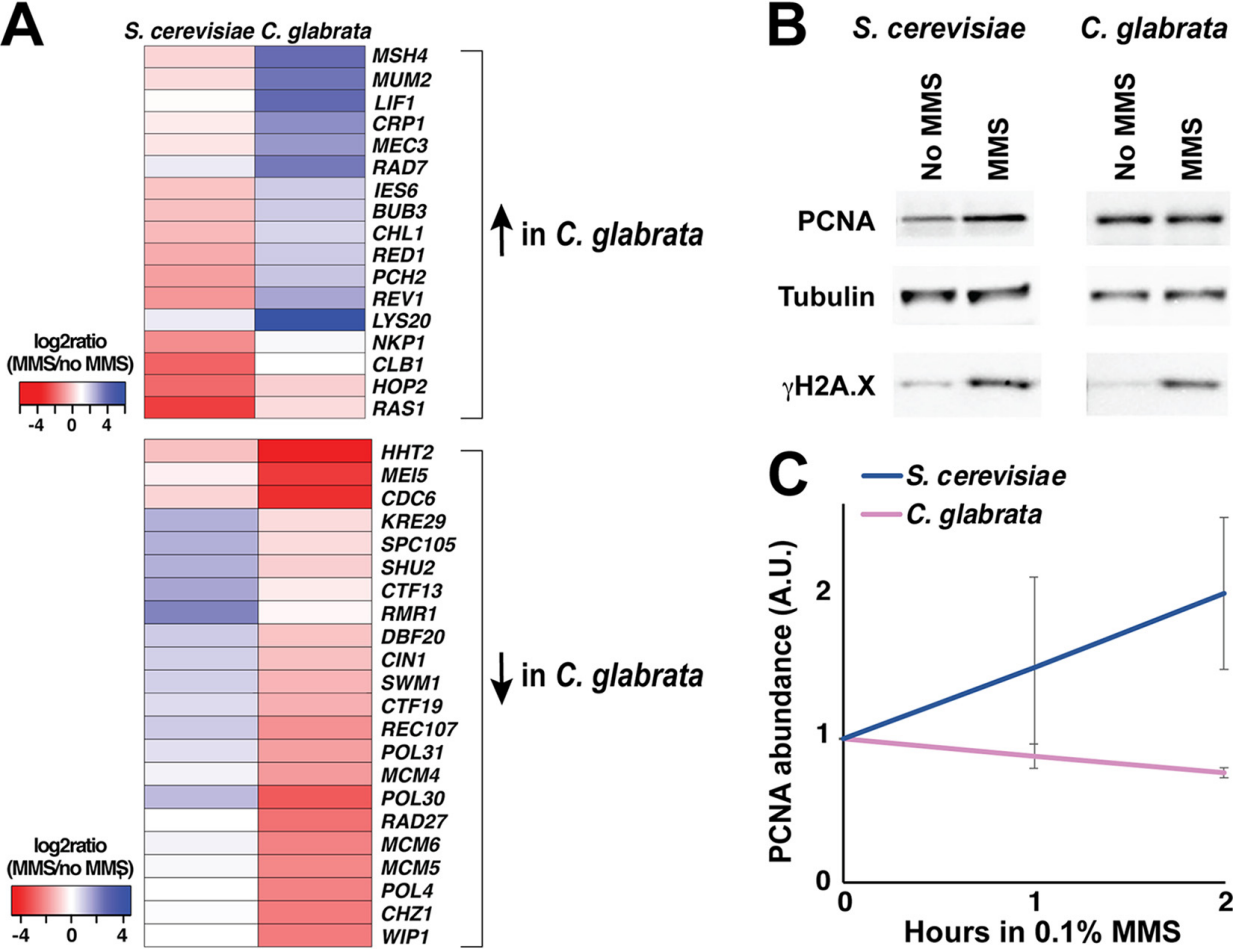
Expression of PCNA is upregulated by DNA damage in S. cerevisiae but downregulated in C. glabrata. (A) Heatmaps of genes involved in maintenance of genome stability and differentially regulated by DNA damage in S. cerevisiae and C. glabrata. (B) PCNA protein levels increase in response to DNA damage in S. cerevisiae but not in C. glabrata. Cells were treated by 0.1% MMS by 2 h and then harvested for total cell lysates and Western blotting. Tubulin, alpha-tubulin. (C) Quantification of Western blot data from at least three biological replicates (Image J). For every condition, PCNA abundance (in arbitrary units [A.U.]) was normalized to that of alpha-tubulin.

We were particularly interested in differentially regulated genes involved in DNA metabolism and genome stability and identified 17 such genes that were upregulated in C. glabrata relative to S. cerevisiae and 22 such genes that were downregulated in C. glabrata relative to S. cerevisiae (Fig. 6A). Interestingly, the latter set contained a number of genes involved in the initiation and progression of DNA replication, including PCNA (*POL30*), a subunit of the prereplicative complex (*CDC6*), several subunits of the MCM (minichromosome maintenance protein complex) replicative helicase, a subunit of DNA polymerase delta (*POL31*), and Okazaki fragment processing exonuclease (*RAD27*). Several of these factors also play key roles in maintaining the stability of DNA replication forks in the presence of DNA damage, most notably PCNA, which mediates multiple interactions between the replisome and various DNA repair complexes (59, 60). *POL30* transcript abundance was induced over twofold by MMS in S. cerevisiae, consistent with other studies (38, 43), but repressed by over eightfold in C. glabrata (Data Set S1). Because a decrease in PCNA abundance is expected to drastically affect the stability of DNA of the replisome, especially in the presence of DNA damage, we sought to confirm that PCNA expression was affected not only at the mRNA level but also at the protein level. Indeed, we found that upon MMS treatment, PCNA abundance increased in S. cerevisiae but decreased in C. glabrata, consistent with RNAseq results (Fig. 6B and C). Together, these results showed that several genes with key roles in maintaining replication fork integrity, including PCNA, are upregulated in S. cerevisiae but downregulated in C. glabrata in response to DNA damage, with likely profound implications on genome stability.

## DISCUSSION

Our study presents the first examination of the DNA damage checkpoint in the opportunistic fungal pathogen C. glabrata. Although C. glabrata is closely related to S. cerevisiae, we found a number of important differences between the DNA damage responses of the two organisms. Unlike S. cerevisiae, C. glabrata did not induce Rad53 phosphorylation or accumulate in S phase upon DNA damage, indicating reduced activation of DNA replication checkpoints. Consistent with attenuated checkpoint signaling, C. glabrata exhibited higher lethality in the presence of DNA damage, and time-lapse microscopy detected evidence of aberrant mitoses under these conditions. Finally, we obtained evidence of diverged transcriptional responses to DNA damage in C. glabrata and S. cerevisiae, including differential regulation of some key protectors of replication integrity, such as PCNA. Together, these results reveal a new variation in eukaryotic DNA damage responses and expand our understanding of factors influencing fungal genetic stability, evolution, and emergence of antifungal drug resistance.

Mechanistic studies in S. cerevisiae have shown that upon DNA damage or replication fork stalling, Rad53 is recruited by adaptor proteins (Rad9 or Mrc1) to activated DNA damage sensor kinases (Mec1/Tel1), which phosphorylate Rad53 at both canonical (S/TQ) and noncanonical sites (35, 61, 62). According to current models, phosphorylated Rad53 accumulates at the sites of DNA damage, further extensively autophosphorylates in *trans*, and then diffuses away to phosphorylate multiple downstream targets (36, 38, 39, 63–66). Interestingly, we found most S/TQ sites are conserved between ScRad53 and CgRad53, suggesting that Mec1/Tel1 phosphorylation of CgRad53 probably occurs and plays an important role. Another piece of evidence suggesting that Mec1 is likely active in C. glabrata is the observed extensive DNA damage-induced phosphorylation of histone H2A-Ser129 (γH2A.X), which is phosphorylated by Mec1 at the sites of damage (67, 68). In contrast, a number of ScRad53 autophosphorylation sites are not conserved in CgRad53. Thus, it is possible that the initial Mec1-catalyzed phosphorylation of Rad53 takes place in C. glabrata but that it does not lead to the same type of autophosphorylation and activation of this effector kinase and consequently does not trigger the same degree of checkpoint activation.

We observed DNA damage-triggered induction or repression of most C. glabrata genes whose S. cerevisiae orthologs are dependent on Rad53, suggesting that these C. glabrata genes are still under checkpoint control. However, a lack of CgRad53 DNA damage-induced phosphorylation suggests that in C. glabrata these genes may not be regulated by Rad53. Indeed, Rad53/CHK2 is not the only DNA damage checkpoint effector kinase in eukaryotic cells. Chk1 (CHK1 in higher eukaryotes) is another serine/threonine effector kinase, which although it plays a minor role in S. cerevisiae, has an important role in the DNA damage and replication checkpoint responses of higher eukaryotes and S. pombe (69). Another effector kinase expressed by yeast cells is Mek1 (meiotic effector kinase), which in S. cerevisiae is meiosis specific and involved in sensing the number of double-strand breaks (DSBs), channeling their repair to promote the appropriate level and distribution of crossovers between homologous chromosomes, and delaying entry into meiosis I until DSB repair has been completed (70). Interestingly, and possibly relatedly, we have detected an upregulation of meiosis and sporulation genes upon DNA damage in C. glabrata. This observation is intriguing because mating and sporulation have not been detected in C. glabrata, although genomic studies suggest that they do happen, albeit extremely rarely (11, 71). Also, interestingly, both CHK1 and MEK1 are transcriptionally upregulated more strongly in C. glabrata than in S. cerevisiae by DNA damage, whereas *RAD53* is similarly and very moderately upregulated in both (see Data Set S1 in the supplemental material). Any possible roles of Chk1 and Mek1 effector kinases in the DNA damage response of C. glabrata will be elucidated in further studies.

In S. cerevisiae, Rad53 is phosphorylated not only by DNA damage sensor kinases Mec1 and Tel1 but also by two cell cycle regulators, cyclin-dependent kinase Cdc28/Cdk1 and Polo-like kinase Cdc5, which phosphorylate ScRad53 at three proline-directed sites (Ser175, Ser375, and Ser774) (51, 52). Interestingly, we found that none of these three phospho-acceptor amino acids are conserved in CgRad53. This lack of conservation is difficult to interpret at present, however, because the role of this phosphorylation in S. cerevisiae is still unclear. On the one hand, alanine substitution mutations at these sites do not cause defects in DNA damage-induced Rad53 phosphorylation or DNA damage sensitivity (51, 52); on the other, phosphorylation of all three of these residues is induced by MMS (35, 62). These alanine substitutions have a few reported phenotypes, including accelerated cellular recovery from a persistent DNA damage checkpoint signal (52) and defects in cell wall integrity (51). The latter may be important in C. glabrata, as its cell wall is the principal mediator of its interaction with the host and a target of antifungal drugs (72, 73). Interestingly, a role of checkpoint proteins in morphogenesis and cell wall integrity has been reported in S. cerevisiae (74). Thus, it will be of interest to examine the role these factors play in cell wall maintenance, drug resistance, and virulence in C. glabrata.

Our transcriptome analysis identified a number of genes involved in maintaining genome stability differentially regulated in S. cerevisiae and C. glabrata. In this study, we focused on PCNA for a number of reasons. First, its transcription was strongly differentially affected by MMS, being induced by over twofold in S. cerevisiae and repressed by over eightfold in C. glabrata. The induction of PCNA expression by DNA damage in S. cerevisiae, which has been reported before and shown to be dependent on ScRad53 (38), is not surprising. Whereas PCNA was originally defined as the processivity factor for DNA polymerases, it is now known to regulate virtually every aspect of chromosomal maintenance, including DNA replication, recombination, repair, and chromatin structure (reviewed in references 59 and 60). *POL30* is an essential gene in S. cerevisiae, but a number of mutant alleles have been generated and shown to exhibit aberrant DNA damage repair and elevated rates of mutation and recombination, among other defects (75–78). A “Decreased Abundance by mRNA Perturbation” (DAmP) *POL30* allele has also been generated, and while its phenotype with respect to genome stability has not been described, large-scale genetic analyses suggest that it behaves similarly to null mutants in nonessential DNA replication genes, such as *RAD27*, *POL32*, and *ELG1* (79). Further studies are necessary to understand why C. glabrata suppresses PCNA expression at a time when it appears to be especially critical for repair of DNA damage and preventing mutagenesis and genomic instability. We also note that it is possible that some PCNA protein abundance differences between C. glabrata and S. cerevisiae at 2 h of MMS exposure (Fig. 6B and C) are due to indirect effects, such as cell cycle differences (Fig. 3). However, PCNA mRNA abundance was also strongly increased in S. cerevisiae but decreased in C. glabrata in cells harvested 1 h after MMS exposure (see, e.g., Fig. 5 legend or Materials and Methods), at which point there were no cell cycle differences between the two yeasts, as both organisms were still predominantly in G_1_ (Fig. 3). Thus, it is likely that the primary reason for the difference in PCNA abundance under these conditions between C. glabrata and S. cerevisiae is alternative transcriptional regulation by DNA damage, with cell cycle differences possibly being a contributing factor.

Both pathogenic and nonpathogenic fungi are characterized by extensive genetic diversity and ability to adapt to new environments (80, 81). In fungal species that can associate with humans, this adaptability is important for microevolution within the host and can translate into the development of drug-resistant infections (5, 8, 82, 83). Evolution of drug resistance in fungal pathogens can be facilitated by acquisition of mutator phenotypes, e.g., due to loss-of-function mutations in DNA mismatch repair genes, as has been observed in clinical *Cryptococcus* and C. glabrata strains (84–86). In diploid or polyploid fungi, such as C. albicans and C. neoformans, environmental stress associated with passage through a mammalian host or antifungal drug exposure leads to increased genetic instability, most notably aneuploidies and loss of heterozygosity (LOH) (3, 4, 7). Because C. glabrata is haploid, it cannot avail itself of these mechanisms. Furthermore, C. glabrata appears to propagate almost exclusively clonally, so it also cannot use meiotic recombination to promote genetic diversity. Yet, C. glabrata genome analyses indicate the occurrence of frequent chromosomal rearrangements (11–13, 24), and our study suggests that these rearrangements may be facilitated by a “lax” DNA damage checkpoint mechanism. C. glabrata is the first obligate haploid commensal/pathogenic fungus whose checkpoint activity has been examined. Thus, it will be of interest to examine whether other haploid fungi, for example Candida auris, which is likewise characterized by extensive genetic variability and high prevalence of antifungal drug resistance (10), may also have a noncanonical DNA damage checkpoint. Finally, although such noncanonical checkpoint mechanisms may facilitate genome instability and emergence of drug-resistant strains, they may also present an exploitable therapeutic opportunity to selectively target checkpoint-deficient cells. For instance, strategies are being evaluated for treating checkpoint-deficient human cancers where it may be possible to inhibit CHK1 in CHK2-deficient cancers or vice versa (87). It would be of interest to investigate similar approaches in fungi, especially those with noncanonical checkpoint responses.

## MATERIALS AND METHODS

### Yeast strain growth and handling.

Common C. glabrata reference strain ATCC 2001 (also known as CBS138) and S. cerevisiae strain W4069-4C (*MAT***a**, W303 genetic background, gift of the Rothstein lab) were used for all experiments. Cells were cultured in standard rich medium (yeast extract-peptone-dextrose [YPD]). S. cerevisiae cells were grown at 30°C and C. glabrata cells were grown at 37°C, which are the optimal growth temperatures for these organisms. To rule out the effects of temperature on cell cycle progression in the presence of DNA damage, we performed this experiment with C. glabrata both at 37°C and 30°C and observed no differences (Fig. 3; see also Fig. S4 in the supplemental material).

### Western blotting.

Whole-cell lysates were prepared by trichloroacetic acid (TCA) precipitation. Briefly, cell pellets were resuspended in 20% TCA, broken by bead beating, and washed twice with 5% TCA, and then proteins were pelleted and resuspended in sodium dodecyl sulfate-polyacrylamide gel electrophoresis (SDS-PAGE) loading buffer. Samples were incubated at 95°C for 5 min and centrifuged prior to loading on acrylamide gels; 8% gels were used to detect Rad53 and 12% gels were used to detect histone H2A, α-tubulin, and PCNA. The following antibodies were obtained commercially: anti-ScRad53 (Abcam ab150018), anti-H2A (Active Motif catalog no. 39945), anti-γH2A.X (Abcam ab15083), anti-PCNA (Abcam ab221196), and anti-α-tubulin (ThermoFisher Scientific catalog no. MA1-80189). Rabbit antibodies against an N-terminal peptide ( IPIKDMEVDVEQIA) and a C-terminal peptide ( GIPNEERSVTSQTE) of CgRad53 were raised by GenScript (GenScript USA Inc., Piscataway, NJ). To help detect CgRad53 by Western blotting, C. glabrata *RAD53* open reading frame (ORF) was subcloned into plasmid pCN-EGD2 (obtained from Addgene) downstream of the weak *EGD2* promoter (48). Cells carrying the resulting plasmid were processed for Western blotting as described above.

### Rad53 phosphorylation analysis by MS.

Rad53 was immunoprecipitated from S. cerevisiae and C. glabrata total cell lysates using the anti-Rad53 antibodies described in the previous section and protein A magnetic beads (New England Biolabs). The immunoprecipitated samples were run on 8% acrylamide gels, stained with GelCode Blue reagent (ThermoFisher Scientific), whereupon an area of roughly 1 cm × 1 cm around the Rad53 band was excised and sent for MS analysis at the Georgetown University Proteomics Shared Resource facility (https://lombardi.georgetown.edu/research/sharedresources/pmsr/proteomics), where the samples were destained and subjected to in-gel proteolytic digestion with trypsin/Lys-C mixture (Promega). The digests were extracted, analyzed by nanoscale ultraperformance liquid chromatography coupled to tandem mass spectrometry (nanoUPLC-MS/MS) using the TripleTOF 6600 mass spectrometer, and mass spectra were recorded with Analyst TF 1.7 software. Data files were submitted for simultaneous searches using Protein Pilot version 5.0 software (Sciex) utilizing the Paragon and Progroup algorithms and the integrated false discovery rate (FDR) analysis function. MS/MS data were searched against either ScRad53 or CgRad53 protein databases, as appropriate. Phosphorylation emphasis was chosen as a special factor. The proteins were inferred based on the ProGroup algorithm associated with the ProteinPilot software. The detected protein threshold in the software was set to the value that corresponded to 1% FDR. All peptides were filtered with confidence to 5% FDR, with the confidence of phosphorylation sites automatically calculated. The Georgetown University Proteomics Shared Resource facility then provided a list of recovered peptides, their intensities, and their posttranslational modifications to the investigators, who used it to calculate the relative abundance of phosphorylated peptides in every sample.

### Cell cycle analysis.

To synchronize S. cerevisiae and C. glabrata in the G_1_ phase of the cell cycle, exponentially growing YPD cultures were shifted to YP medium (no dextrose) and cultured for 18 h. At that point, cells were resuspended in YPD in the absence or presence of 0.03% MMS or 100 mM HU and cultured for another 6 h. Aliquots were fixed with 70% ethanol at every hour. Prior to analysis by flow cytometry, the samples were pelleted and resuspended in phosphate-buffered saline (PBS), sonicated, and treated with an RNase cocktail (Fisher Scientific). The cell counts in each sample were measured and adjusted to the same cell concentration, followed by addition of SYTOX Green (ThermoFisher Scientific) and flow cytometric analysis using the BD Fortessa instrument.

### Cell viability measurements.

To calculate the percentage of viable cells in cultures containing MMS, the drug was added to exponentially growing C. glabrata or S. cerevisiae cultures at desired concentrations. At various time points thereafter, aliquots were removed, cells were counted using hemocytometer slides, and plated on drug-free YPD plates. Percentage viability was calculated based on the observed numbers of colonies relative to the corresponding cell counts.

### Fluorescence microscopy.

The NLS-RFP construct was subcloned from plasmid pML85 (gift of Michael Lisby) into C. glabrata plasmid pMJ22 (88) (obtained from Addgene) using XhoI and NotI restriction sites. Slides for time-lapse microscopy were prepared by pipetting warm YPD containing 1% low-melting-point agarose (with or without 0.03% MMS) onto glass slides and letting it solidify, forming YPD-agarose pads. Exponentially growing C. glabrata cells carrying the NLS-RFP plasmid were pipetted onto the YPD-agarose pads, covered with coverslips, and sealed using Biotium coverslip sealant (Fisher Scientific). The cells were imaged at room temperature for 6 h at 10-min intervals using a Nikon Eclipse Ti2 inverted microscope and Hamamatsu ORCA-Flash4.0 camera and analyzed using NIS-Elements software.

### Transcriptome analysis.

Exponentially growing C. glabrata and S. cerevisiae cells were exposed to 0.1% MMS for 1 h, at which point cells were harvested and total RNA was extracted using the RNeasy kit (Qiagen). The RNA samples were sent to Genewiz (South Plainfield, NJ) for RNAseq. Three biological replicates for each condition were submitted, but one “S. cerevisiae no MMS” sample failed quality control and was not processed further. The RNAseq data were analyzed using Base pair software (Basepair, New York, NY) with a pipeline that included the following steps. Reads were aligned to the transcriptome derived from sacCer3 using STAR with default parameters. Read counts for each transcript was measured using featureCounts. Differentially expressed genes were determined using DESeq2, and a cutoff of 0.05for the adjusted *P* value (corrected for multiple hypotheses testing) was used for creating differentially expressed gene lists. GSEA was performed on normalized gene expression counts, using gene permutations for calculating *P* value. Raw RNAseq data files have been deposited at the Gene Expression Omnibus (accession no. GSE155701). The list of C. glabrata*-*S. cerevisiae direct orthologs was downloaded from http://www.candidagenome.org/download/homology/orthologs and supplemented by manual curation of C. glabrata genes using http://www.candidagenome.org. Gene Ontology (GO) analysis was performed using FungiFun (https://elbe.hki-jena.de/fungifun/) (89). Heatmaps were generated using R studio.

### Data availability.

All processed data are available as Data Set S1 in the supplemental material. Raw RNAseq data are available from the Gene Expression Omnibus (https://www.ncbi.nlm.nih.gov/geo/query/acc.cgi?acc=GSE155701).
