# Supplementary figures and images for: A Noncanonical DNA Damage Checkpoint Response in a Major Fungal Pathogen
Source: mBio. 2020 Dec 15;11(6):e03044-20. doi: 10.1128/mBio.03044-20 (PMC7773997; doi:10.1128/mBio.03044-20)

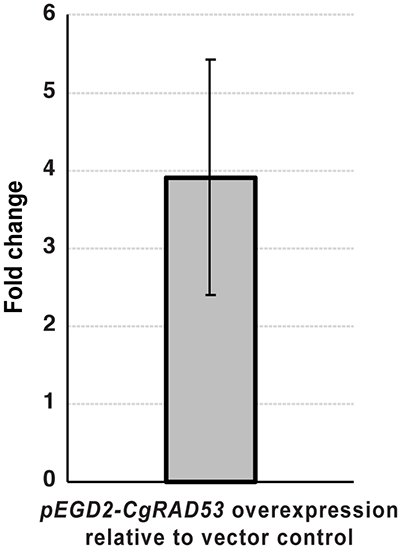

Supplement: FIG S1 [file mBio.03044-20-sf001.tif]

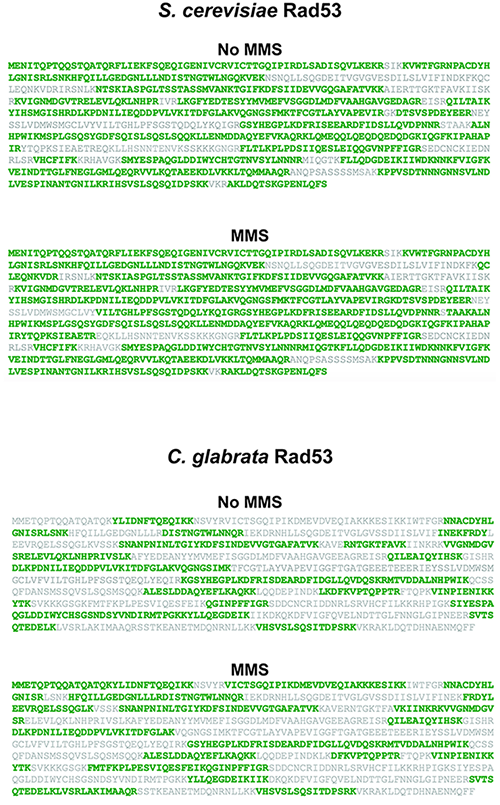

Supplement: FIG S2 [file mBio.03044-20-sf002.tif]

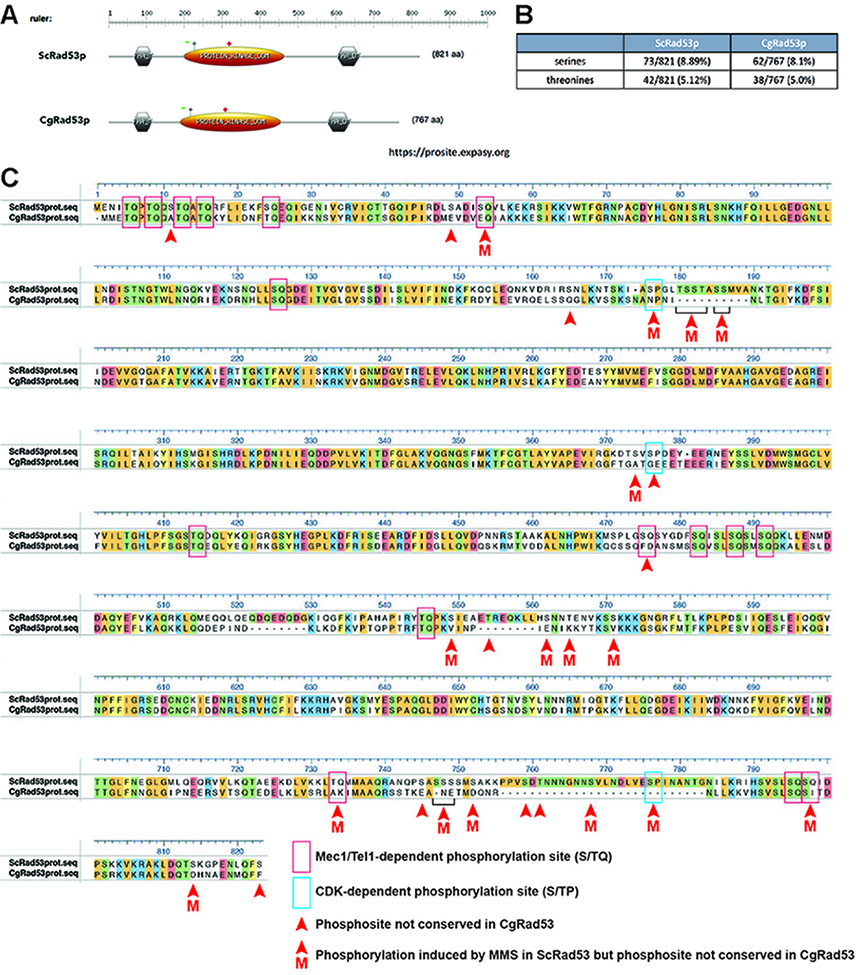

Supplement: FIG S3 [file mBio.03044-20-sf003.tif]

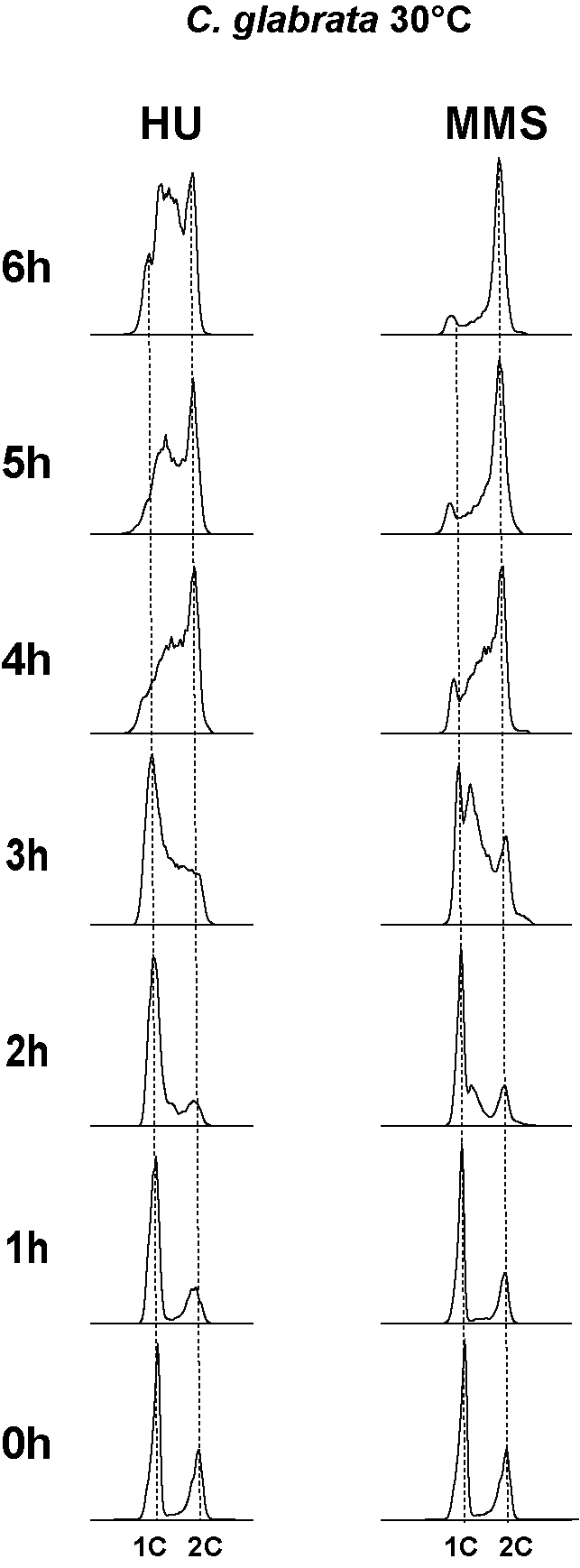

Supplement: FIG S4 [file mBio.03044-20-sf004.tif]

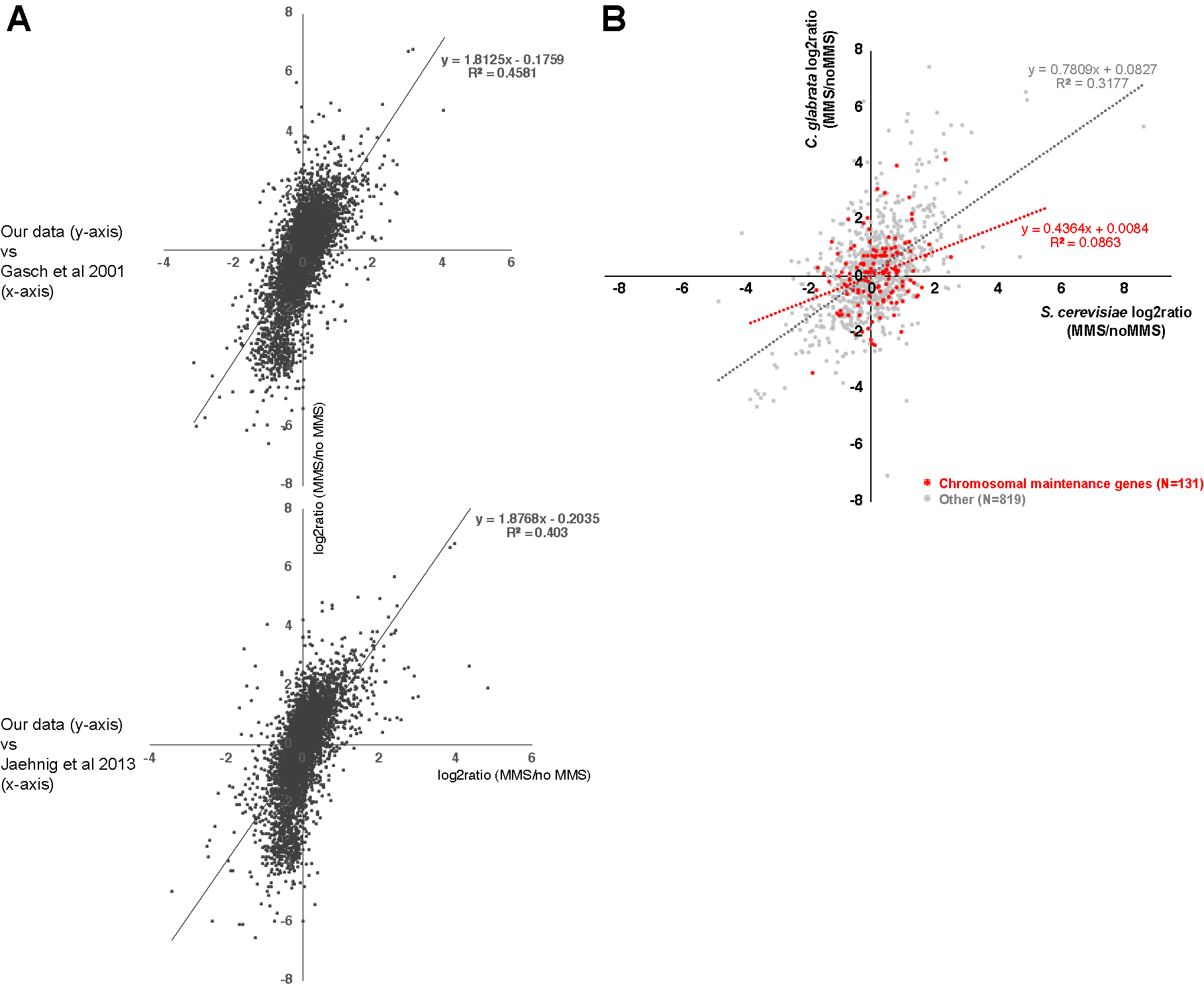

Supplement: FIG S5 [file mBio.03044-20-sf005.tif]

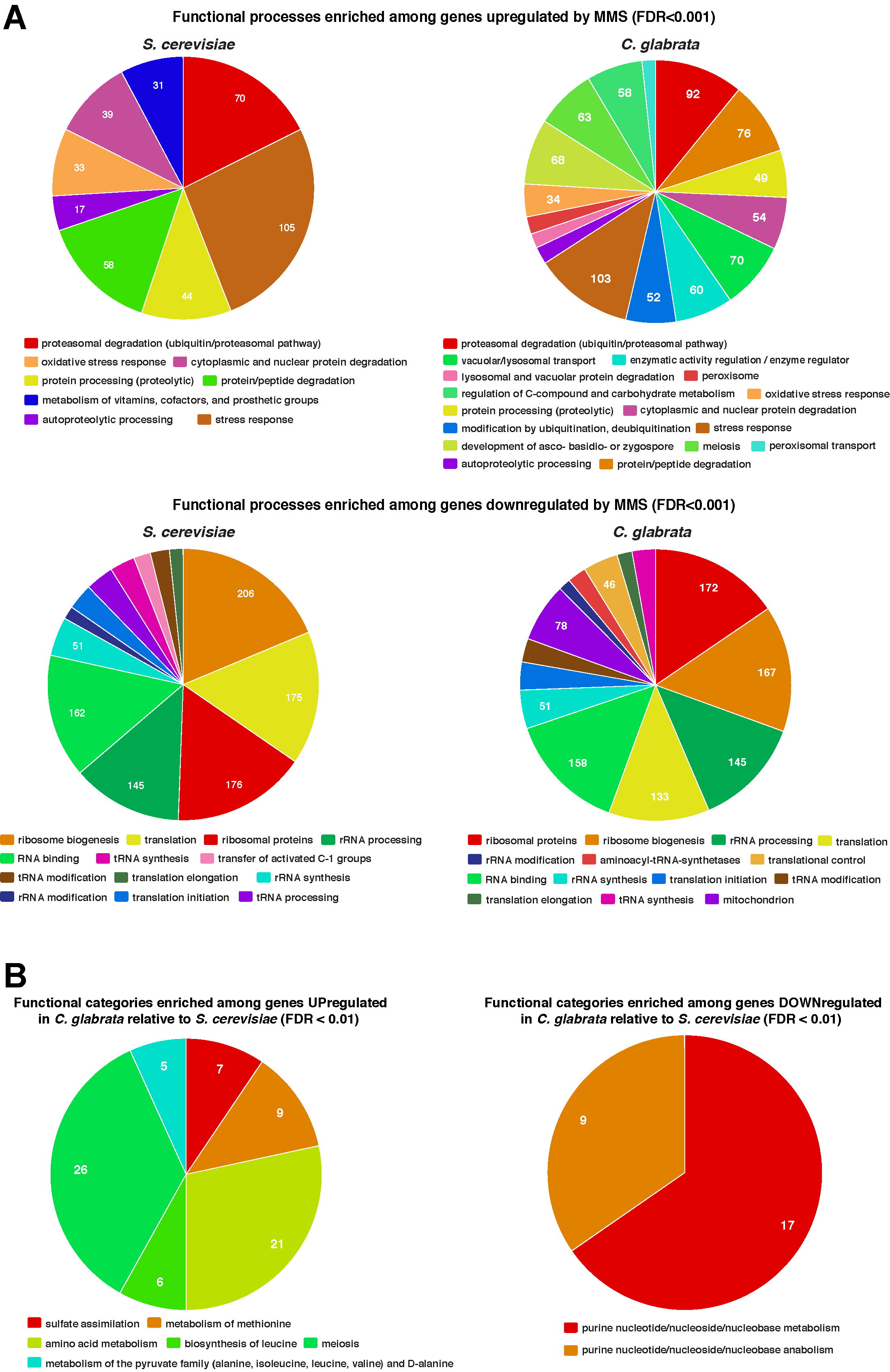

Supplement: FIG S6 [file mBio.03044-20-sf006.tif]
